# Identification of upstream regulators for prognostic expression signature genes in colorectal cancer

**DOI:** 10.1186/1752-0509-7-86

**Published:** 2013-09-04

**Authors:** Taejeong Bae, Kyoohyoung Rho, Jin Woo Choi, Katsuhisa Horimoto, Wankyu Kim, Sunghoon Kim

**Affiliations:** 1 College of Pharmacy, Seoul National University, Seoul 151-742, South Korea; 2 Information Center for Bio-pharmacological Network, Seoul National University, Suwon 443-270, South Korea; 3 Medicinal Bioconvergence Research Center, Advanced Institutes of Convergence Technology, Suwon 443-270, South Korea; 4 DNA Link Inc, Seoul, South Korea; 5 Wonkwang Institute of Interfused Biomedical Science, Wonkwang University, Seoul 150-827, South Korea; 6 Department of Pharmacology and Wonkwang Institute of Dental Research, School of Dentistry, Wonkwang University, Iksan, Chonbuk 570-749, South Korea; 7 Computational Biology Research Center, National Institute of Advanced Industrial Science and Technology, Tokyo 135-0064, Japan; 8 Ewha Research Center for Systems Biology (ERCSB), Ewha Womans University, 52 Ewhayeodae-gil, Seodaemun-gu, Seoul 120-750, South Korea; 9 World Class University Program Department of Molecular Medicine and Biopharmaceutical Sciences, Seoul National University, Seoul 151-742, South Korea

**Keywords:** Gene signature, Colorectal cancer, Transcriptional network, Network inference

## Abstract

**Background:**

Gene expression signatures have been commonly used as diagnostic and prognostic markers for cancer subtyping. However, expression signatures frequently include many *passengers*, which are not directly related to cancer progression. Their upstream regulators such as transcription factors (TFs) may take a more critical role as *drivers* or master regulators to provide better clues on the underlying regulatory mechanisms and therapeutic applications.

**Results:**

In order to identify prognostic master regulators, we took the known 85 prognostic signature genes for colorectal cancer and inferred their upstream TFs. To this end, a global transcriptional regulatory network was constructed with total >200,000 TF-target links using the ARACNE algorithm. We selected the top 10 TFs as candidate master regulators to show the highest coverage of the signature genes among the total 846 TF-target sub-networks or regulons. The selected TFs showed a comparable or slightly better prognostic performance than the original 85 signature genes in spite of greatly reduced number of marker genes from 85 to 10. Notably, these TFs were selected solely from inferred regulatory links using gene expression profiles and included many TFs regulating tumorigenic processes such as proliferation, metastasis, and differentiation.

**Conclusions:**

Our network approach leads to the identification of the upstream transcription factors for prognostic signature genes to provide leads to their regulatory mechanisms. We demonstrate that our approach could identify upstream biomarkers for a given set of signature genes with markedly smaller size and comparable performances. The utility of our method may be expandable to other types of signatures such as diagnosis and drug response.

## Background

With advances in genome-wide gene expression technologies, classification of cancer subtypes based on expression signatures is widespread and results in many biomarkers for various cancers. This molecular signature-based approach is more objective and reproducible than conventional methods based on clinicopathological features. There are plenty of clinical applications that are actively being sought [1-3]. Some of these are already in commercial use [4,5] for selecting treatment strategies and predicting prognosis. In spite of the advantages and successful applications, the identification of causal oncogenic pathways and driver-regulators remains a challenge [6]. The main bottleneck is that expression signatures normally consist of cancer drivers and passengers with the latter as not directly related to cancer progression. The reason for this is that passengers frequently take the majority of the signature gene and an accurate discrimination of cancer drivers from passengers becomes a key subject in cancer genomic studies.

Regulatory network modeling has been widely used for a systematic understanding of disease progression at the molecular level, particularly for cancer (comprehensively reviewed by Peer and Hacohen) [7]. Recently, Carro et al. applied a reverse engineering method for context-specific transcriptional regulatory networks to 176 gene expression profiles from high-grade glioblastoma (HGG) patients. Two TFs (C/EBPβ and STAT3) were successfully identified as master regulators and control ‘mesenchymal’ signature genes that lead to tumor aggressiveness such as epithelial-to-mesenchymal transition and neo-angiogenesis [8]. They applied the ARACNE algorithm for global reconstruction of regulatory network [9], where directed or causal TF-target relationship was extracted from measuring conditional mutual information. Then, the regulatory TFs for the mesenchymal signature genes were inferred from the use of master regulator analysis (MRA) together with or without stepwise linear regression method (SLR). This provides an exemplary case to pinpoint upstream regulators of known cancer signatures as cancer drivers and, accordingly, to a promising therapeutic target. Further, this strategy also provides a chance to develop biomarkers of even smaller sizes than the original signature, which is highly desirable for practical usage in terms of cost and interpretation.

In this study, we used Carro et al. [8] as the framework of our analysis and applied the same method to colorectal cancer with only minor modifications. Colorectal cancer is one of the most commonly diagnosed cancers and the fourth leading cause of cancer-related death in males and the third in females worldwide [10]. Several research groups have identified prognostic molecular signatures that use genome-wide gene expression profiles of colorectal cancer patients [11-13]. Recently, Oh et al. classified 177 colorectal patients into two groups from the use of global gene expression profiles and extracted 85 signature genes (114 probe set) that were differentially expressed between the two groups. This gene signature shows a good prognostic ability to discriminate colorectal cancer patients between good and poor prognostic groups with high accuracy [14]. We reasoned that the upstream regulators or transcription factors (TFs) of these prognostic signatures might take a critical role as *driver* or master regulator to provide clues on the underlying regulatory mechanisms and therapeutic applications. Here, we applied a reverse engineering algorithm to reconstruct an unbiased transcriptional network from colorectal cancer. Using this network, the upstream regulators of the prognostic signatures were identified and tested for their utility as prognostic markers. Our network models provide clues on the potential regulatory mechanisms for these upstream regulators that may cause prognostic differences.

## Results and discussion

### Overview of the analytic procedure

Our analytic procedure followed that of Carro et al. [8]. In the study, a global regulatory network was inferred from high-grade glioblastoma (HGG). The difference was that we focused on modeling regulatory networks only for the 85 prognostic marker genes in colorectal cancer reported by Oh et al. [14]. From the use of the network model, we then extracted their upstream regulators or TFs and tested their prognostic ability in comparison with the original 85 signature genes.

**Figure 1:**
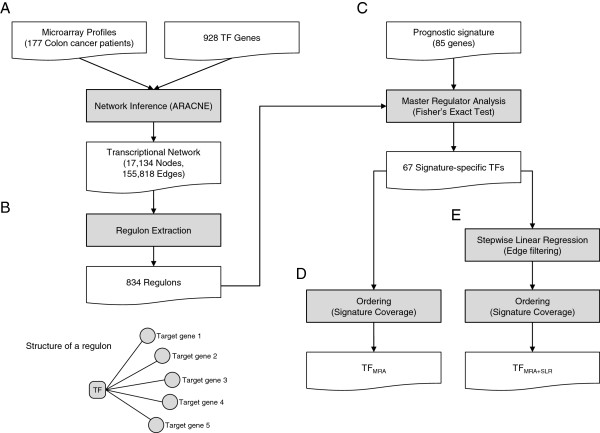
**Overall pipeline of upstream regulator inference. (A)** Global regulatory network modeling using ARACNE. **(B)** Regulon extraction for each TF. **(C)** Master regulator analysis (MRA) selects the TFs showing a significant overlap with the prognostic signature genes **(D)** Extraction of top 10 TFs by the signature coverage of MRA derived regulons **(E)** Stepwise linear regression (SLR) for edge filtering and extraction of top 10 TFs by the signature coverage of MRS + SLR derived regulons.

As the detail of mathematical formulation is described from the previous work [9] and the methods section, we briefly summarize our overall procedures (Figure 1). Once a global regulatory network was constructed using the ARACNE algorithm, regulons or TF targets are extracted for all candidate TFs. Top candidate TFs were chosen based on the coverage of signatures as downstream regulated genes (= regulons). This procedure or *master regulator analysis* (MRA) is equal to conventional gene set analysis (GSA) based on the Fisher exact test. Alternatively, we applied a stepwise linear regression method (SLR) for each signature gene and its expression was modeled using a minimal set of candidate TFs. In our case, SLR was used only to filter out weak TF-target relations in each regulon and to keep the most obvious interactions modeled by simple linear equations. In contrast, Carro et al. expanded the candidate TFs before the application of SLR by including additional 52 TFs with their promoter sequences enriched among the signature genes [8]. Therefore, our study is more suitable to evaluate whether a regulatory model can successfully identify key upstream regulators (e.g. prognostic markers) purely based on expression profiles without depending on external knowledge.

### Construction of regulatory networks and identification of upstream regulators for prognostic signatures

First, we took the 177 expression profiles from colon cancer patients from Moffit Cancer Center (Moffit cohort, n = 177 [12]). They were also used to extract the 85 prognostic signature genes for colorectal cancer. Then the ARACNE algorithm was applied to infer a global transcriptional network. In total, we inferred 155,818 TF-target interactions between 834 TFs and 17,065 target genes in the context of colorectal cancer (Figure 1A). In total, 834 regulons were extracted, each consisted of a TF and its target genes (Figure 1B). For the 834 regulons, we applied MRA, which tests significant overlap between the regulons and the 85 signature genes (Figure 1C). MRA identified 67 TFs, of which targets significantly overlap with the signature at a false discovery rate (FDR) < 0.05 (Additional file 1: Table S1). The 67 TFs collectively regulate 84 of the 85 signature genes (Figure 1D).

We further applied SLR to the regulons identified by MRA. In this step, the expression level of each signature gene was modeled by the linear combination of the expression levels of its upstream TFs in the network. The reason that the SLR method tries to minimize the number of TFs in modeling the expression level of each signature gene is that only the TFs showing strong linear correlation tend to remain in the final regression model. Accordingly, SLR was essentially used as a filtering step to remove less effective TF-target interactions (Figure 1E).

**Figure 2:**
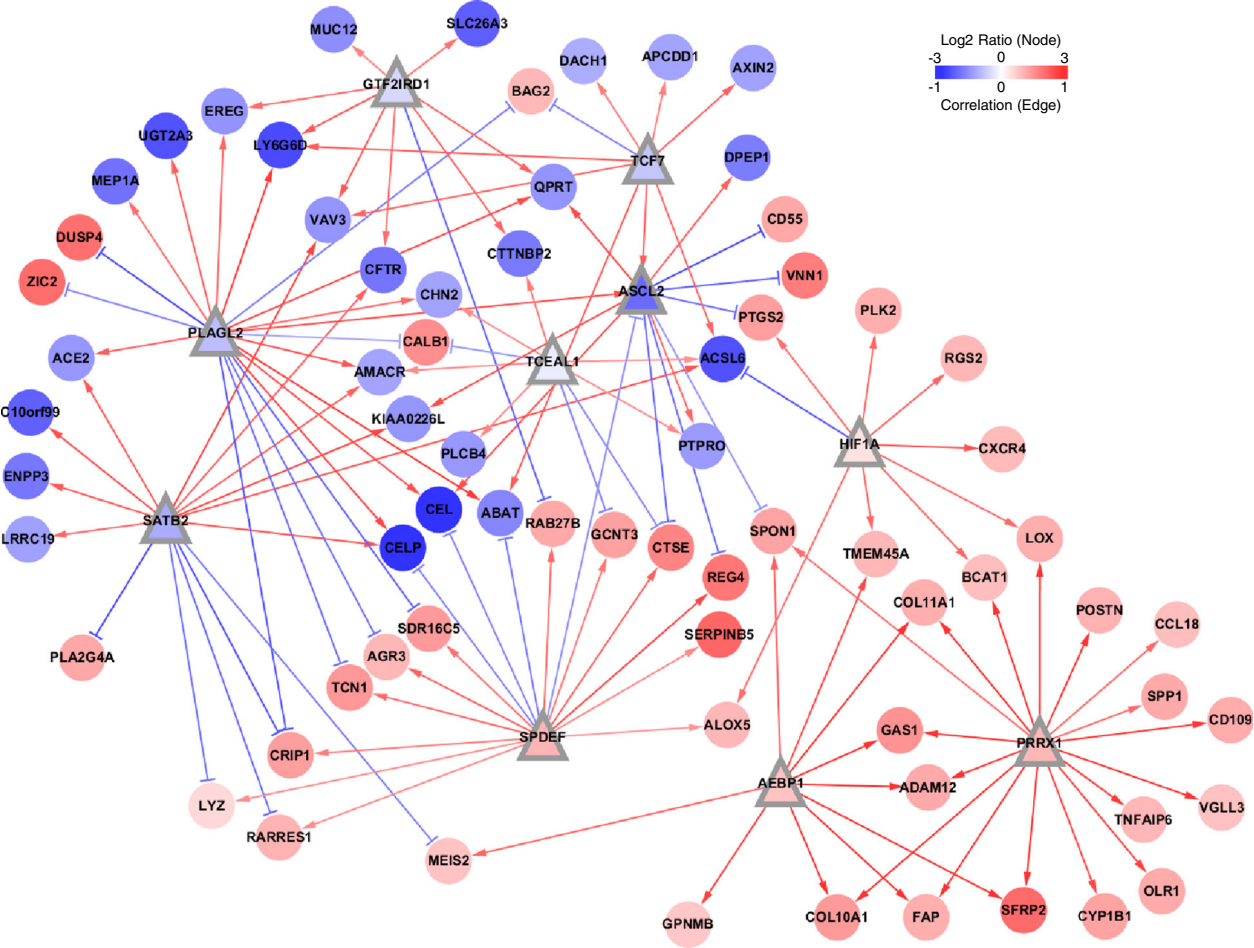
**The transcriptional network between the top 10 TFs and the signature genes by MRA + SLR method.** Node shape is triangular for TFs and circle for target signature genes. Node color represents the log2 ratio of gene expression between the poor and the goop prognostic group in the Moffit cohort (n = 177). Arrow shapes represent regulatory modes determined by the sign(+/−) of Spearman’s rank correlation between a TF and its target gene. Edge color represents the magnitude of correlation.

The TFs were ranked by the order of signature coverage, i.e. the number of signature genes regulated by corresponding TF. In MRA and MRA + SLR method, the first 10 TFs covered most of the 85 signature genes. In MRA, the coverage the top 10 TFs was 83 out of the 85 signature genes with the average number of target genes per TF = 8.3. In case of MRA + SLR, the coverage was 71 out the 85 genes with 7.1 target genes per TF. These two sets of top 10 TFs by MRA and MRA + SLR method were chosen as candidate upstream regulators for further analysis and named as TF_MRA_ and TF_MRA+SLR_, respectively (Table 1). The two TF sets largely agreed to each other with 7 TFs in common (i.e. PLAGL2, PRRX1, SPDEF, SATB2, ASCL2, HIF1A, and TCF7). Three TFs were specific for MRA (BCL6, TFCP2L1, and FOSL2) and MRA + SLR (AEBP1, GTF2IRD1, and TCEAL1), respectively. We constructed two versions of regulatory networks between the top 10 upstream regulators (TF_MRA_ and TF_MRA+SLR_) and their downstream targets among the 85 signature genes. Additional file 1: Table S4 lists the downstream signature genes of each TF. Figure 2 and Additional file 2: Figure S1 visualizes networks for TF_MRA_ and TF_MRA+SLR_. Notably, some transcription factors were linked by positive or negative regulatory interactions. ASCL2 was positively regulated by two TFs (PLAGL2 and TCF7) and negatively by SPDEF to suggest a higher order structure among the upstream regulators. Many of the prognostic signature genes were co-regulated by several TFs, e.g. ACSL6 by four TFs (TCF7, TCEAL1, SATB2, and HIF1A) and VAV3 by three TFs (GTF2IRD1, SATB2, and TCF7).

**Table 1:** **Overall statistics of 13 TFs, union of TF**_**MRA **_**and TF**_**MRA+SLR**_

**TF symbol**	**Prognostic effect**	**Regulon size**	**MRA**^**1**^			**MRA + SLR**^**2**^	
			**Rank**	**Signature coverage**	**FDR**^**3**^	**Rank**	**Signature coverage**
PLAGL2	+	575	1	32	7.73E-25	1	20
PRRX1	-	327	4	22	2.10E-18	2	17
SPDEF	-	304	6	18	6.50E-14	3	16
SATB2	+	264	3	23	1.28E-21	4	15
ASCL2	+	537	2	28	6.12E-21	5	11
AEBP1	-	465	15	12	2.21E-05	6	10
TCF7	+	408	9	15	9.47E-09	7	9
TCEAL1	+	276	16	10	1.08E-05	7	9
HIF1A	-	371	4	22	2.58E-17	7	9
GTF2IRD1	+	429	16	10	0.000334	7	9
BCL6	-	455	7	16	4.50E-09	11	8
TFCP2L1	+	364	9	15	2.26E-09	14	7
FOSL2	-	421	7	16	1.70E-09	19	6

^1^Master Regulatory Analysis.

^2^Stepwise Linear Regression.

^3^False Discovery Rate.

### Prognostic effect analyses for the upstream regulators identified by MRA and MRA + SLR

The 85 signature genes consisted of 34 low-risk and 51 high-risk marker genes that were significantly up and down-regulated, respectively, in the patient group of better survival [14]. Accordingly, we assigned the prog-nostic effect of the 67 TFs as positive (+) or negative (−) class that depends on whether the majority of the downstream target genes are regulated in favor of expressing low-risk or high-risk signatures. First, we calculated Spearman’s rank correlation between each TF and its downstream signature genes. The regulatory mode was determined by the sign of Spearman’s rank correlation between a TF and its target, where positive correlation indicated ‘activation’ and negative did ‘repression’. The prognostic effect of a TF was assigned positive (+) if the sum of activated low-risk and repressed high-risk genes was more than half among its downstream signature genes. Among the 67 TFs selected by MRA, the prognostic effect of the 30 TFs was positive with the remaining 37 TFs being negative (Additional file 1: Table S3).

**Figure 3:**
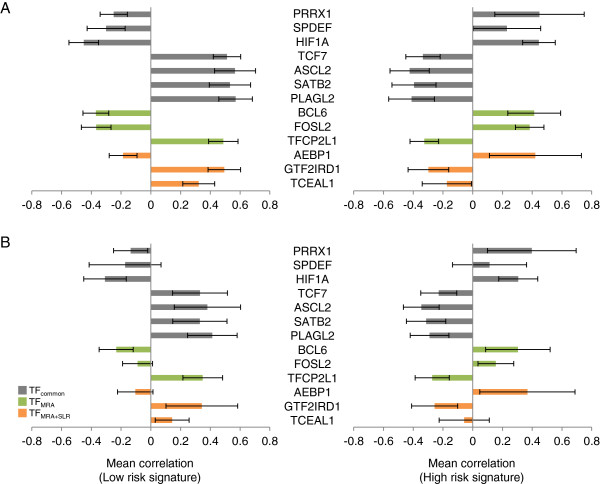
**Correlation between the upstream TFs and their target genes.** The average of the Spearman’s rank correlation coefficients was calculated between each of the 13 TFs (union of TF_MRA_ and TF_MRA+SLR_) and the low risk (left) or the high risk (right) signature genes for **(A)** Moffit cohort and **(B)** Melbourne cohort.

We focused on the top 10 TFs in TF_MRA_ and TF_MRA+SLR_ and asked whether their prognostic effect is consistently observed across different data sources. However, the Moffit cohort used for network construction by ARACNE, we took another set of gene expression profiles from Royal Melbourne Hospital (Melbourne cohort, n = 95) [11]. Positive prognostic effect was observed in five out of the 10 TFs in TF_MRA_ and four in TF_MRA+SLR_ in the Moffit cohort (Figure 3A). The rest five and six TFs showed negative prognostic effect, respectively. We observed exactly the same trend for all the TFs tested in the Melbourne cohort to suggest that their regulatory interactions were consistently maintained in colorectal cancer (Figure 3B).

### Strong association of the top 10 upstream TFs with the survival of colon cancer patients

**Figure 4:**
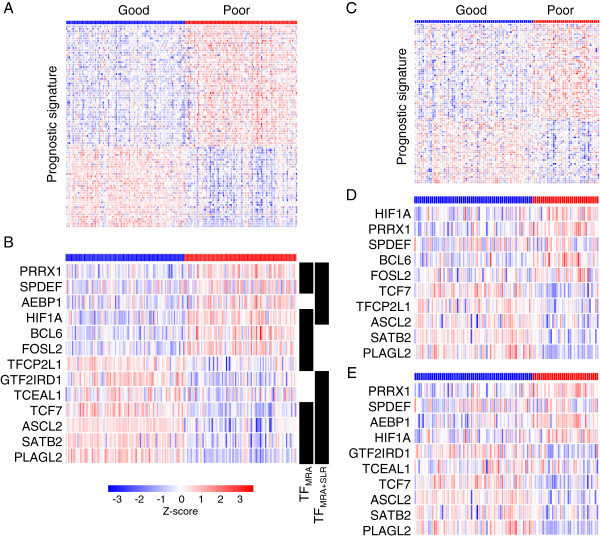
**Expression patterns of the selected marker genes between the good and the poor prognostic group.** The distinct expression pattern of **(A)** 85 signature genes and of **(B)** 13 TFs (union of TF_MRA_ and TF_MRA+SLR_) are shown in the Moffit court (n = 177, training dataset). Differential expression pattern is observed to be well maintained in an independent test dataset (Melbourne cohort, n = 95) for **(C)** the 85 signature genes, **(D)** TF_MRA,_ and **(E)** TF_MRA+SLR_ after class prediction.

Now, we tested the utility of the upstream regulators (TF_MRA_, TF_MRA+SLR_) as prognostic markers for colorectal cancer. In the Moffit cohort (n = 177) used as the training dataset, TF_MRA_ and TF_MRA+SLR_ showed a strong differential expression pattern between good and poor prognostic groups similar to the original 85 signature genes (Figure 4A, 4B). An SVM (support vector machine) classifier was constructed for TF_MRA_, TF_MRA+SLR_, and the original 85 signature genes. For validation purposes, we took the Melbourne cohort (n = 95) as an independent test set. These 95 patients were classified into *good* or *poor* prognostic groups independently from the use of each of the three classifiers. For all three classifications, the resulting good and poor prognostic groups showed the same differential expression patterns in the test dataset (Figure 4C, 4D, and 4E).

**Figure 5:**
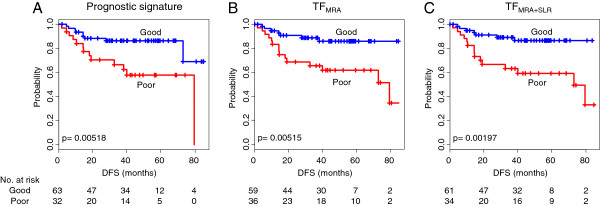
**The prediction performance of the selected prognostic markers.** Kaplan-Meier plots for disease-free survival (DFS) are shown between the good and the poor prognostic group for **(A)** the 85 signature genes, **(B)** TF_MRA_, and **(C)** TF_MRA+SLR_. P-value for difference between two K-M plots was calculated by log-rank test.

We compared the prognostic performance of the three classifiers using the Kaplan-Meier plots for disease-free survival (Figure 5). The upstream TFs showed a slightly better or similar performance than the original 85 signature genes with the ordering of TF_MRA+SLR_ > TF_MRA_ > 85 signature genes. The p-values by log-rank test were 1.97×10^-3^ for TF_MRA+SLR_, 5.15×10^-3^ for TF_MRA_, and 5.15×10^-3^ for the 85 signature genes. We further inspected the prognostic performance over a range of signature sizes for the gene signatures as well as the TF signatures (Additional file 2: Figure S2). Overall, a stable prognostic utility was observed over a range of signature sizes for both TF-based methods (P < 0.05 using 10 ~ 38 TFs by MRA and 7 ~ 21 TFs by MRA + SLR). Although the best prognosis was observed for the gene signature of size = 18 ~ 20, the TFs showed a reasonably good performance comparable to the 85 signature genes using the top 7–11 TFs by MRA + SLR and 10 ~ 19 TFs by MRA. Notably, these upstream TFs were not selected directly for good (or poor) survival but by the coverage of known prognostic signatures in our regulatory network model based purely on expression profiles. Therefore, the performances of TF_MRA_ and TF_MRA+SLR_ are thought to be unexpectedly high, considering that the signature size dramatically decreased to less than 1/8 (from 85 to 10 genes) to demonstrate that upstream TFs can be even better prognostic markers than the expression signatures. The same strategy may be useful in identifying upstream regulators for other types of cancer signatures such as drug response and metastatic behavior.

### Candidate upstream regulators include many TFs involved in tumorigenesis: HIF1A FOSL2, PLAGL2, ASCL2, and TCF7

Many of the upstream TFs for the prognostic signature genes are actually well known regulators for various tumorigenic processes such as cell invasion, metastasis, and clinical outcome. Among the TFs of poor prognostic effect, HIF1A and FOSL2 are examples of such cases. Our network models also recapitulate some of the known TF-target relations, as confirmed by the literature. Hypoxia-inducible factors (HIFs) are the key regulators of oxygen signaling pathway that respond to oxygen-deficient environment known as hypoxia. Cancer cells overcome hypoxic conditions by hypoxic pathway activated by HIFs. HIF1A is overexpressed in a variety of human cancers and is associated with poor prognosis in various cancers [15,16] including colon cancer [17]. Among the nine targets of HIF1A in our network by MRA + SLR, the three interactions are confirmed by the literature. HIF1A activates CXCR4 and LOX and are involved in metastasis in renal cell carcinoma [18] and hypoxia-induced metastasis [19], respectively. PTGS2 (known as COX2) is known to be directly up-regulated by HIF1A and promotes hypoxia-induced angiogenesis [20]. In addition, PTGS2 is shown negatively regulated by ASCL2, one among the top 10 TFs in both networks. FOSL2 (also known as FRA2) is a member of FOS family, which encodes leucine zipper proteins forming AP-1 transcription factor complex together with JUN family proteins. While FOSL2 is included in the top 10 TFs only in TF_MRA_, its rank is still relatively high in TF_MRA+SLR_ (19th out of the 67 TFs). FOSL2 is known to mediate cell growth and differentiation [21] and its transgenic mice show a severe loss of small blood vessels in skin [22] to suggest a role in angiogenesis. FOSL2 also activates LOX in our network by MRA (Additional file 2: Figure S1).

Among the TFs of good prognostic effect, PLAGL2 is notable due to its dual functionality as proto-oncogene and tumor suppressor. PLAGL2 has been known as a proto-oncogene in acute myeloid leukemia (AML), glioblastoma (GBM), and colorectal cancer [23-25]. PLAGL2 can activate Wnt signaling that leads to leukemia in mice [23] or suppression of cellular differentiation [25]. Contrarily, PLAGL2 also functions as tumor suppressor that promotes apoptosis or arrests cell cycle [26-28]. ASCL2 and TCF7 (also known as TCF-1) are TFs activated by Wnt signaling. ASCL2 is up-regulated in colorectal adenocarcinoma [29] and, until recently, growth arrests are observed by knockdown of ASCL2 in vivo [30]; although the prognostic effect of ASCL2 was positive (+). TCF7 is a member of the TCF/LEF family, which transmit the Wnt signal into the nucleus and activate Wnt target genes by interacting with β-catenin. Unlike other members of TCF/LEF family, TCF7 may act as negative regulators for Wnt signaling because its isoforms lack a β-catenin binding domain, while retaining Groucho interaction domain necessary for repressor activity [31,32].

There is evidence that tumorigenic activity for other TFs such as PRRX1 (PMX1) and SPDEF (PDEF). The gene fusion between PRRX1 and NUP98 was reported in AML [33]. Suppressive activities for metastasis, cell growth, and migration are suggested for SPDEF [34,35].

## Conclusions

We propose a genetic analysis pipeline to find transcriptional modules for prognostic gene expression signatures or other biomarkers. Our method only requires expression profiles in the appropriate context such as tissue type or disease condition. This procedure was applied to identify key upstream regulators for the 85 prognostic signature genes for colorectal cancer. To this end, a global transcriptional network was constructed using the ARACNE algorithm [9]. Candidate upstream regulators were selected based on the number of signature genes as downstream targets or regulons (MRA step). An additional filter was applied to extract only strong TF-target interactions readily modeled by simple linear regression (SLR step). As a result, we identified two sets of top 10 TFs that clearly discriminate between good and poor prognostic groups. The prognostic performance was tested using a dataset independent of signature selection and network modeling. These upstream TFs included many known regulators for tumorigenic processes such as metastasis and cell proliferation. The utility of our work is two-fold. The first is that it allows the identification of upstream regulators for a given set of signature genes and provides leads to regulatory mechanisms. The second is that these regulators may serve as better biomarkers by themselves than the original signature with markedly smaller sizes and better performance. The utility of our method may be expandable to other types of signatures such as diagnosis and drug response.

## Methods

### Data set

The 85 prognostic signature genes for colorectal cancer were obtained from S-C Oh et al., which was derived by mapping the 114 probes to the corresponding genes [14]. The gene expression profiles from the Moffit cohort (GSE17536, n = 177) and those from the Melbourne cohort (GSE14333, n = 95 after removal of redundancy) were obtained from Gene Expression Omnibus database (http://ncbi.nlm.nih.gov/geo). All the expression profiles used were generated using Affymetrix HG-U133 Plus2.0 GeneChip array. The raw CEL files were processed and normalized using the MAS5 method (affy package in R/Bioconductor). The list of TFs was obtained from Carro et al. [8] and includes 928 human TFs. These TFs were mapped to 2155 probe sets in Affymetrix HG-U133Plus2.0 GeneChip array.

### Network inference using ARACNE

ARACNE (http://wiki.c2b2.columbia.edu/califanolab/index.php/Software/ARACNE) was used to infer interactions between the 2155 TF probe sets and their target genes. The gene expression profiles of the Moffit cohort were used in this analysis. Threshold for MI (mutual information) and DPI (Data Processing Inequality) tolerance were set to p < 0.05 (Bonferroni corrected for multiple testing) and 0%, respectively. The bootstrapping option was applied to generate 100 bootstrapped networks. These networks were merged into a consensus network from consensus voting methods based on a statistically significant number of interactions inferred from the bootstrapping steps. As probe sets in network were mapped to genes, the consensus network was merged into the gene level network.

### Master regulator analysis

Fisher’s exact test was used to determine statistical significance for overlaps between target genes in each regulon. The FDRs for the p-values were computed using procedures described by Benjamini and Hochberg [36]. Then, the signature-enriched TFs were ranked by signature coverage, which is the edge number linked with signature genes.

### Stepwise linear regression analysis

A linear model for each signature gene was constructed as follows. The log2-expression level of TFs linked to each signature gene was considered as the explanatory variables. The log2-expression level of each signature gene was considered as the response variable. Then, we used stepwise algorithm in order to select the best minimal set of the explanatory variables in each model. Akaike information criterion (AIC) was used as the stop criterion. TFs with a p-value for linear regression coefficient that was less than 0.05 were removed in selected variables.

### Class prediction and survival analysis

BRB-Array Tools (http://linus.nci.nih.gov/BRB-ArrayTools.html) was used for building SVM classifier and class prediction. The survival package in R was used for Kaplan-Meier plot and log-rank test.

## Abbreviations

MRA: Master regulator analysis; SLR: Stepwise linear regression; AML: Acute myeloid leukemia; GBM: Glioblastoma; TCF: T-cell factor; LEF: Lymphoid enhancer factor.

## Competing interests

The authors declare that they have no competing interests.

## Author contributions

TB and WK participated in the design of the study, conducted computational and statistical analysis, as well as wrote the manuscript. KR participated in the design of the study and supervised computational and statistical analysis. JWC helped interpret the results in perspective of colorectal cancer biology. KH helped to check the validity of the overall pipeline used for computational and statistical perspectives. SK supervised and funded the entire study. All authors read and approved the final manuscript.

## Supplementary Material

Additional file 1 This file includes the list of the prognostic signature genes of colorectal cancer, the list of all TFs selected by MRA, detailed summary of prognostic effect of those TFs and the edge list of the transcriptional network of the top 10 TFs by MRA and MRA + SLR method. Click here for file

Additional file 2: Figure S1 The transcriptional network between the top 10 TFs and the signature genes by MRA method. **Figure S2.** The influence of the signature size on the prognostic performance of the gene signature (blue), TF_MRA_(green), and TF_MRA+SLR_(orange). The 85 signature genes were ordered by the fold change degree of differential expression between the two groups in the original publication publication [14]. The TFs were ordered by the coverage of the 85 signature genes in the regulons. The signature genes or TFs were sequentially included by the corresponding order and the prognostic performance was measured by p-values using Kaplan-Meier plot. Click here for file
