# Supplementary material for: Identification of upstream regulators for prognostic expression signature genes in colorectal cancer
Source: BMC Syst Biol. 2013 Sep 4;7:86. doi: 10.1186/1752-0509-7-86 (PMC3847874; doi:10.1186/1752-0509-7-86)
Supplement: Additional file 2: Figure S1 — The transcriptional network between the top 10 TFs and the signature genes by MRA method. Figure S2. The influence of the signature size on the prognostic performance of the gene signature (blue), TFMRA(green), and TFMRA+SLR(orange). The 85 signature genes were ordered by the fold change degree of differential expression between the two groups in the original publication publication [14]. The TFs were ordered by the coverage of the 85 signature genes in the regulons. The signature genes or TFs were sequentially included by the corresponding order and the prognostic performance was measured by p-values using Kaplan-Meier plot. [file 1752-0509-7-86-S2.pptx]

## Slide 1
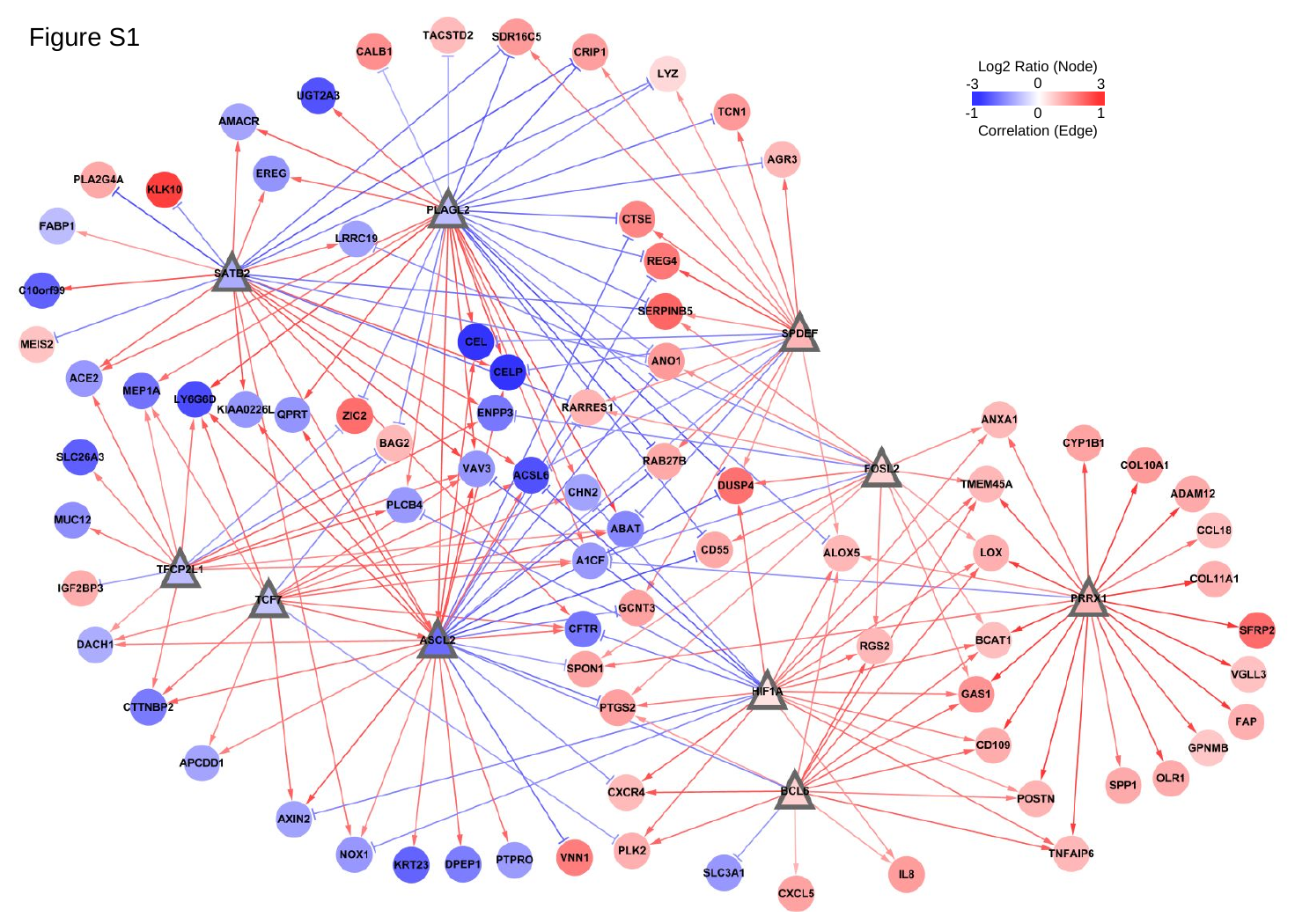

Figure S1
Log2 Ratio (Node)
0
-3
3
-1
0
Correlation (Edge)
1

## Slide 2
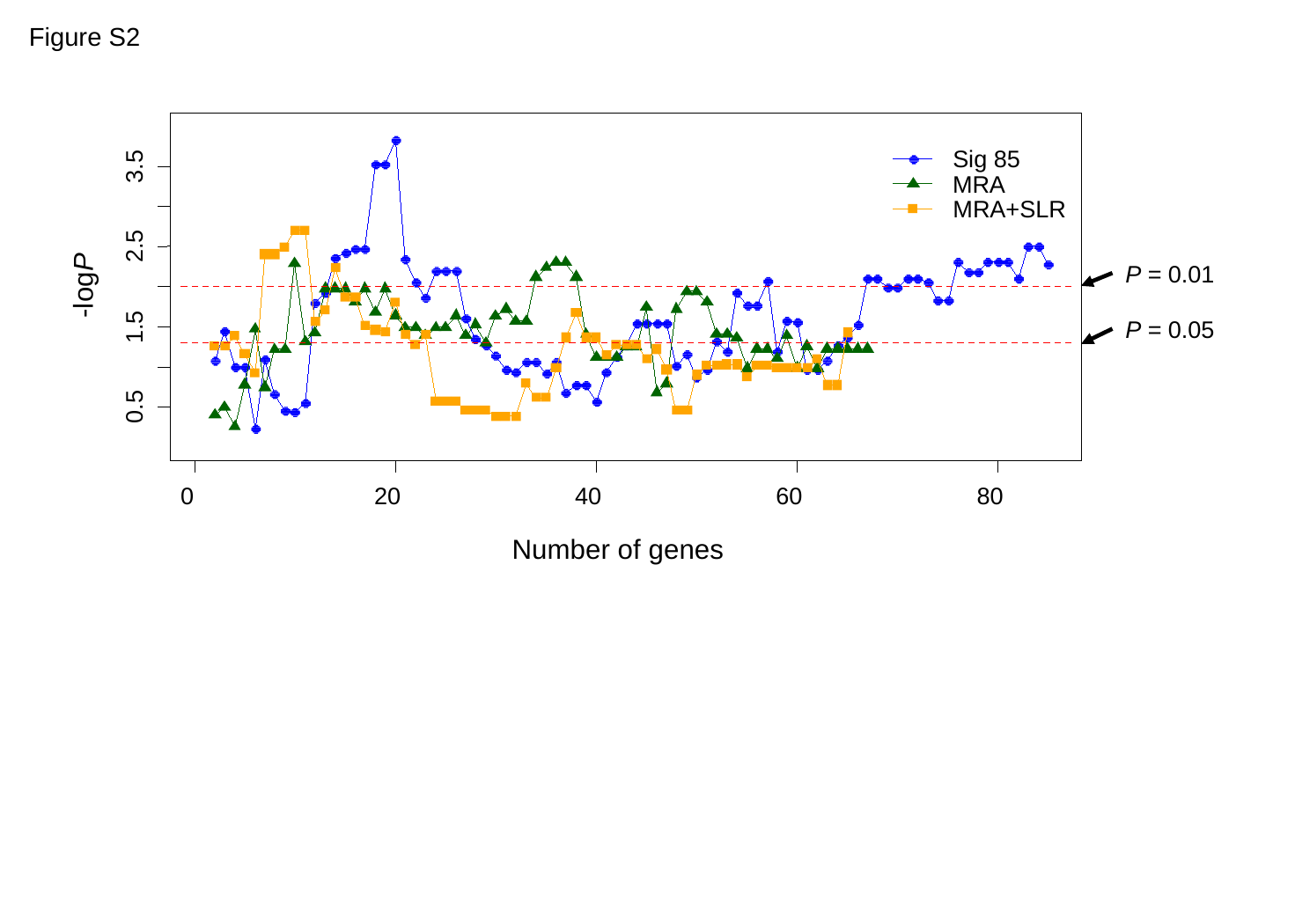

Figure S2
3.5
2.5
-logP
1.5
0.5
0
20
40
60
80
Number of genes
Sig 85
MRA
MRA+SLR
P = 0.01
P = 0.05
